# Bilateral Open Achilles Tendon Complete Lacerations Following Interpersonal Violence: A Case Report

**DOI:** 10.1155/cro/5557419

**Published:** 2025-06-12

**Authors:** Collen Sandile Nkosi, Bongiwe Hadebe, Papa Kwabena Offeh Kyei

**Affiliations:** Division of Orthopaedic Surgery, University of the Witwatersrand, Johannesburg, South Africa

**Keywords:** laceration, male, open Achilles, orthopedics, trauma

## Abstract

**Introduction:** Interpersonal violence victims account for a sizable proportion of trauma cases encountered in South African trauma centers. Open Achilles tendon injuries are more prevalent in male patients, accounting for a high number of all cases.

**Case Report:** A 45-year-old male was brought in a trauma casualty by a paramedic after being referred by a local clinic in severe pain on both ankles, unable to walk or stand, and dressed bilaterally with blood stains. On examination, he demonstrated the triad of Achilles tendon injuries, including Matle's sign, Thompson's test, and a gap on palpation. A surgical operation was performed within 24 h of the patient's admission.

**Conclusion:** This case report is aimed at increasing awareness of a rare case that presented with open bilateral Achilles tendon injuries associated with an alleged assault and received a successful surgical treatment without a skin complication.

## 1. Introduction

Interpersonal violence victims account for a sizable proportion of trauma cases encountered in South African trauma centers [1]. Community assaults have been defined as unstructured, very violent attacks against suspected perpetrators carried out by members of a neighborhood with the intent of causing catastrophic injuries [1]. The Achilles tendon is the strongest and thickest tendon in the body, created by the joining of the soleus, lateral, and medial gastrocnemius tendons [2]. It has the capacity to support 12 times the body's weight, and in recent years, it accounts for more than 20% of all big tendon injuries, with the number growing globally [3, 4].

Open Achilles tendon injuries are more prevalent in male patients, accounting for 97% of all cases at an age of 32.1 years [5]. The mechanisms of injuries of open Achilles tendon injuries include slippage in the bathroom, a grinder, a road traffic accident, a machete cut, a glass cut, broken bottles, and broken toilet seats [5, 6]. The treatment of open Achilles tendon injuries is not debatable, although it is difficult due to the wound's unique incidence and contamination. Early debridement, antibiotics, and repair can lead to good clinical results [6, 7].

There was no prior literature that we could uncover that documented a case of acute traumatic bilateral open Achilles tendon complete lacerations. We report an atypical case of a healthy male who presented with open bilateral Achilles tendon injuries associated with an alleged assault.

## 2. Case Report

A 45-year-old male was brought in a trauma casualty by a paramedic after being referred by a local clinic for severe pain in both ankles, unable to walk or stand with blood-stained dressings bilaterally. He gave a history of allegedly being assaulted with a blunt object on the body and a sharp object on the ankles. He was cleared using advanced trauma life support by the trauma team on call and handed over to the orthopedic senior registrar on call. He denied any past medical history, use of medications, or use of substances.

On examination, he had a 5–7 cm bilateral posterior ankle transverse lacerations with no active bleeding sutured with nylon. The Thompson squeeze test was bilaterally positive, Matle's sign was bilaterally positive, and there were bilateral 4 cm gaps proximal to the insertional site. He had good bilateral foot perfusion with no sensation on the posterolateral aspect of both feet.

**Figure 1:**
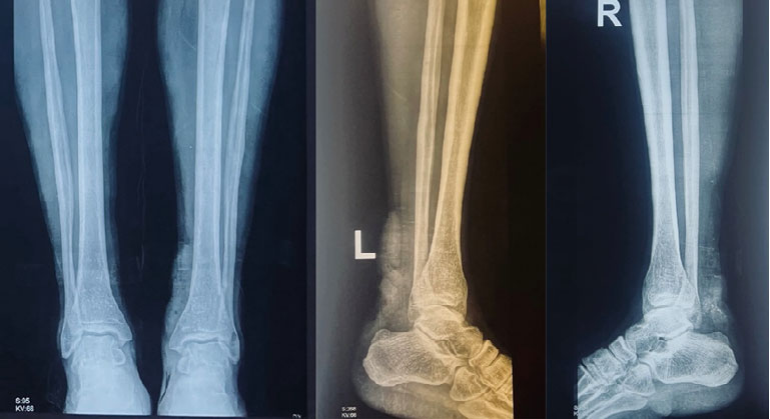
Radiographs of the bilateral ankle anteroposterior and lateral views with disrupted Kager's triangle on the lateral views and with no fractures.

Plain radiographs of the distal legs showed disrupted Kager's triangles on lateral views with no obvious fractures (Figure 1). He had a low hemoglobin level of 9.5 g/dL, and crush injury was cleared.

**Figure 2:**
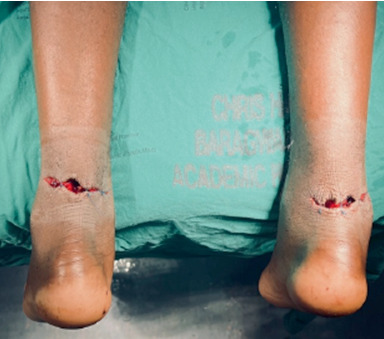
Sutured wounds on the posterior region of the ankles after washout.

In trauma casualty, the lacerations were irrigated with 3 L of normal saline for each wound. Following the removal of the sutures, tetanus prophylaxis was given, and cefazolin intravenous 1 g every 8 h was started for a duration of 3 days. Lacerations were approximated with nylon (Figure 2), dressed with wet gauze, and placed in bilateral anterior slabs in an equinus foot position.

**Figure 3:**
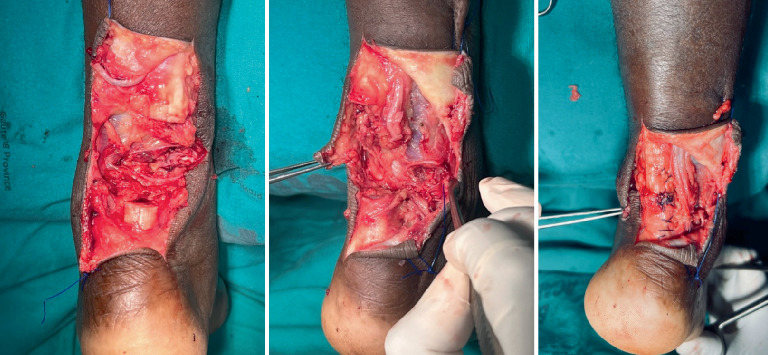
Intraoperative images of the bilateral Achilles tendon completely lacerated and sutured.

Within 24 h of the patient's admission, a surgical procedure was undertaken. The patient was placed in the prone position, and tourniquets were applied before turning the patient to the prone position. The procedures were done under sterile conditions. Posteromedial approaches were used over the transverse lacerations, and the complete lacerations of the Achilles tendons and sural nerves were confirmed (Figure 3).

The wounds were debrided, and end-to-end Achilles tendons were repaired using the Vicryl Krakow suture technique, followed by the continuous suture technique. Paratenons were thereafter repaired. The sural nerves were end-to-end repaired as well. Wound closure was done in layers, and a dry dressing was applied. He was placed in 20° equinus anterior below-knee slabs for 2 weeks and was nonweight bearing. At 2 weeks, sutures were removed, and below-knee full casts were placed for 4 weeks in the 20° equinus position.

At the 6-week review, physiotherapy was initiated with the use of moon boots and wedges using a 10° wedge for a duration of 3 weeks, enabling neutral to plantar flexion motion of the feet. At 9 weeks, he was taken off the moon boots. At 12 weeks, he improved excellently, but there had been no sural nerve recovery. At the 7-month review, his American Orthopaedic Foot and Ankle Society (AOFAS) Ankle-Hindfoot score for both open Achilles tendon injuries was 63/100.

## 3. Discussion

Acute lacerations of the Achilles tendon often result from a sharp instrument penetrating the ankle's posterior region and causing a partial to complete tendon injury. There is a lack of published research on open injuries to the Achilles tendon, and there is not a single case report of bilateral open Achilles tendon in English literature.

To the best of our knowledge, this study represents the first case report on the uncommon occurrence of bilateral open Achilles tendon lacerations. Said et al. reported on over 200 cases that were treated for open Achilles tendon lacerations with unstated sides in the article [8]. In previous studies, the incidence of unilateral open Achilles tendon injury was high among male-to-female cases [5, 8, 9]. The majority of reported cases had experienced open Achilles tendon injuries in their third decade of life [5, 6]. The open unilateral Achilles tendon injuries were caused by an assault with a sharp cutting instrument, a vehicle-related accident, slipping on the restroom floor, shattered sharp objects, and other factors [5, 6].

Unilateral Achilles tendon injuries have been observed to be most frequent in the right leg [7].

Open Achilles tendon injuries appear to be common in the general population; in this case, the patient was a civilian who had sustained bilateral open tendon injuries [6]. The open injuries to the Achilles tendon can be classified as partial or complete, transverse or oblique or crush, single-level or multiple-level, and clean or contaminated [5–7, 10]. The presentation of open injuries differs from closed injuries with bleeding, open wounds, and the mechanism of injury [10].

The open Achilles tendon injuries are diagnosed clinically and confirmed by imaging [4]. In this case, plain radiographs were used to exclude fractures and confirm Kager's triangle on the lateral radiographs [2].

The management of open Achilles tendon injuries is strictly surgical debridement, antibiotics, repair, soft tissue cover, and rehabilitation. In previously published studies, casualty treatment included tetanus, antibiotics, wound irrigation, dressing, and immobilization, with a plan to take the cases to the theatre within 24 h. Theatre treatment included debridement, Achilles tendon repair, and soft tissue reconstruction [6, 10]. Rehabilitation varies depending on the facility. These injuries are not without repercussions. The following challenges are frequently reported: hematomas, venothrombolic events, infections, stitch abscesses, keloid scars, persistent pain, repeated tendon ruptures, sural nerve injuries, skin necroses, wound dehiscence, and others [9–11].

In conclusion, this case report is aimed at increasing the awareness of a rare case that presented with open bilateral Achilles tendon injuries associated with an alleged assault and received successful surgical treatment without skin complications but experienced delayed recovery of bilateral sural nerves postrepair with promising functional outcomes.

## Data Availability

The data that support the findings of this study are available from the corresponding author upon reasonable request.
